# Fish Assemblages on Estuarine Artificial Reefs: Natural Rocky-Reef Mimics or Discrete Assemblages?

**DOI:** 10.1371/journal.pone.0063505

**Published:** 2013-06-03

**Authors:** Heath Folpp, Michael Lowry, Marcus Gregson, Iain M. Suthers

**Affiliations:** 1 NSW Department of Primary Industries, Recreational and Indigenous Fishing Division, Coffs Harbour Jetty, NSW, Australia; 2 NSW Department of Primary Industries, Wild Fisheries Research Division, Port Stephens Fisheries Institute, Nelson Bay, NSW, Australia; 3 University of New South Wales, School of Biological, Earth and Environmental Sciences, Sydney, NSW, Australia; University of California Davis, United States of America

## Abstract

If the primary goal of artificial reef construction is the creation of additional reef habitat that is comparable to adjacent natural rocky-reef, then performance should be evaluated using simultaneous comparisons with adjacent natural habitats. Using baited remote underwater video (BRUV) fish assemblages on purpose-built estuarine artificial reefs and adjacent natural rocky-reef and sand-flat were assessed 18 months post-deployment in three south-east Australian estuaries. Fish abundance, species richness and diversity were found to be greater on the artificial reefs than on either naturally occurring reef or sand-flat in all estuaries. Comparisons within each estuary identified significant differences in the species composition between the artificial and natural rocky-reefs. The artificial reef assemblage was dominated by sparid species including *Acanthopagrus australis* and *Rhabdosargus sarba*. The preference for a range of habitats by theses sparid species is evident by their detection on sand-flat, natural rocky reef and artificial reef habitats. The fish assemblage identified on the artificial reefs remained distinct from the adjacent rocky-reef, comprising a range of species drawn from naturally occurring rocky-reef and sand-flat. In addition, some mid-water schooling species including *Trachurus novaezelandiae* and *Pseudocaranx georgianus* were only identified on the artificial reef community; presumably as result of the reef's isolated location in open-water. We concluded that estuarine artificial reef assemblages are likely to differ significantly from adjacent rocky-reef, potentially as a result of physical factors such as reef isolation, coupled with species specific behavioural traits such as the ability of some species to traverse large sand flats in order to locate reef structure, and feeding preferences. Artificial reefs should not be viewed as direct surrogates for natural reef. The assemblages are likely to remain distinct from naturally occurring habitat comprised of species that reside on a range of adjacent natural habitats.

## Introduction

Artificial reef development in south-east Australia has followed a similar pattern to the evolution of artificial reefs projects worldwide. During the 1960 s, artificial reefs were deployed within estuarine systems as they were cheaper to construct and typically provided proximate and economic access [1]. These early artificial reefs were normally constructed as small patch reefs made from waste material and ‘materials of opportunity’ and deployed in areas of soft-bottom substrate [2], [3]. The failure of these early initiatives to mature into a larger strategy for fisheries enhancement was a result of inadequate knowledge regarding the design and deployment of artificial structures and the lack of post-deployment monitoring which resulted in an inability to demonstrate the potential of artificial reefs to provide local recreational and commercial fisheries enhancement.

More stringent environmental regulations, combined with a growing body of research into how artificial reefs and fish assemblages interact [4]–[8], has resulted in the development of purpose built artificial reefs [9]. In 2005, an estuarine artificial reef project began in south-east Australia with the deployment of a series of small artificial reef in three coastal estuaries using purpose built Reef Ball® modules. The project aimed to provide additional fishing locations for recreational fishers that would be similar in species composition to naturally occurring rocky reef. These trial artificial reef deployments have been followed by renewed interest in the use of artificial reefs in other Australian states (Victoria, Queensland and Western Australia). The rapid expansion and growing interest in the deployment of artificial structures has highlighted the need for information that can guide the development of existing and future artificial reef projects.

Assessing the performance of an artificial reef is dependent on accurately monitoring the fish assemblages at both artificial and suitable naturally occurring habitats [10]. There are inherent issues relating to artificial reefs that make direct comparisons with natural reef difficult. Apart from design dependent physical differences (size, shape and complexity) artificial reefs are, in general, more isolated and younger than naturally occurring habitats [10]. As a consequence, artificial reefs frequently develop fish and benthic communities with abundance and diversity that is comparable to, or greater than, that of nearby natural reefs [11]–[14]. In addition, the majority of existing research relating to fish assemblages associated with artificial reefs and naturally occurring habitats has compared tropical coral reef fish communities [15]–[18] with limited investigations into their use in a temperate estuarine environment.

Most studies of estuarine artificial reefs have focussed on comparisons with non purpose built artificial reef structures such as revetment walls [12], [18] and other coastal infrastructure such as wharves, marinas and swimming enclosures [19]. The results of these studies indicate that the response of fish assemblages associated with artificial structures in estuarine systems is inconsistent. In some cases, artificial structures supported more species than did nearby natural reefs [11], [20]. Other studies have found diversity to be greater around natural reef than around artificial structures [2], [4]. It has also been observed that the type of artificial structure played a significant role in the fish assemblages associated with it. Different artificial structures (e.g. marinas versus swimming enclosures), located in the same area, but which differed in their physical attributes (size and complexity), were shown to have varying species numbers and diversity [19]. Hence, it is unclear if similar patterns in diversity and community structure exist not only between estuaries, but even within estuarine systems. An advantage to using purpose built artificial reefs is that the same structure is deployed across a suite of estuarine and environmental conditions to examine the response by the fish assemblage.

To provide a quantitative comparison of fish assemblages associated with artificial reefs (of a standardised size, age and complexity) with adjacent natural habitats in three south-east Australian estuaries, baited remote underwater video (BRUV) data was selected from a summer to autumn period (Dec – May), 12–18 months post artificial reef construction. The predicted hypothesis based on previous studies that have investigated and compared artificial structure assemblages with those assemblages found on natural reef habitat is that after a minimum of 1 year post artificial reef deployment, the assemblage identified on the artificial reef would be comprised of a similar suite of species to those found on the natural rocky-reef. However the proportional distribution of assemblages associated with artificial reefs when compared to the rocky-reef would vary as an artefact of reef type, location and age. Hence, the aims of the study were to (i) identify dominant species and family groups, and; (ii) investigate the ability of the artificial reefs to support an assemblage comparable to those identified on the natural rocky-reef and to provide surrogate habitat to the broader estuarine fish community.

## Methods

### Ethics statement

All field studies outlined in this paper were authorised under a scientific research permit (permit No: P01/0059) issued by the NSW Department of Primary Industries under section 37 of the Fisheries Management Act 1994. This permit authorises the collection of fish in all waters of NSW. The estuarine sites sampled with baited remote under water video (BRUV) were not privately owned or protected and no endangered or protected species were involved in this study. All fish observations using BRUV was carried out in an ethical manner and no fish were euthanased as part of this study.

### Study sites

**Figure 1:**
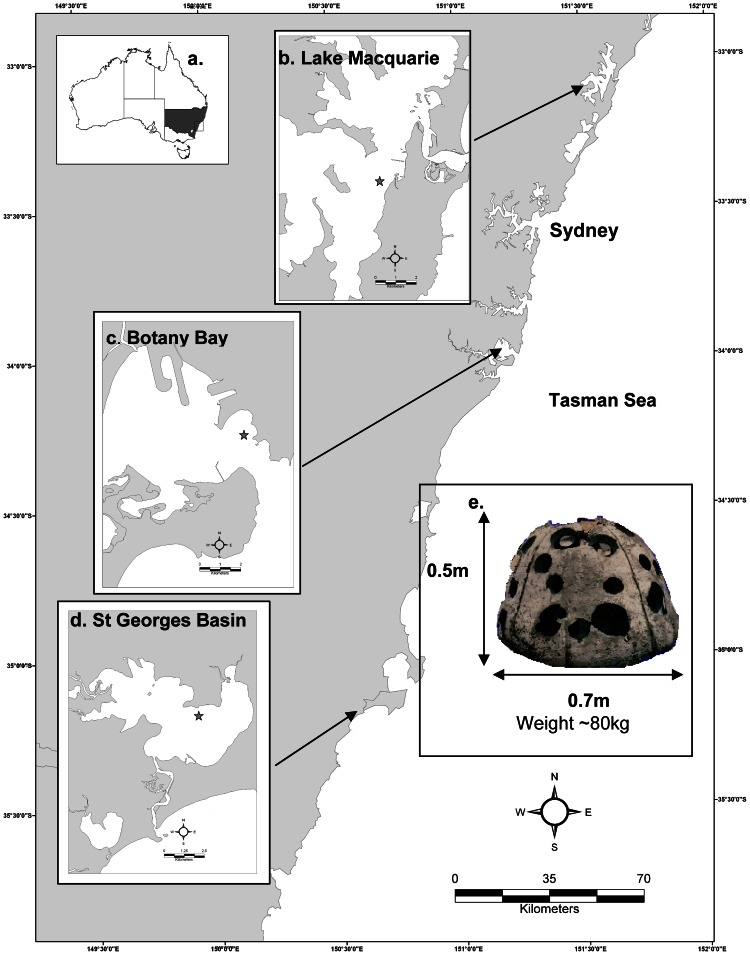
Study locations. A. Locations of the three estuarine study sites; B. Lake Macquarie; C. Botany Bay; D. St Georges Basin – the ‘star’ in each sub-map (A, B, C) represents approximate artificial reef locations; E. Artificial reef module (‘mini-bay’ Reef Ball^®^).

The artificial reefs were constructed as part of a larger study to investigate the use of artificial reef in three coastal estuaries along Australia's south east coast in the State of New South Wales [21]–[24]. Lake Macquarie (33°09' S 151°66' E), Botany Bay (33°00' S 151°23' E) and St Georges Basin (35°18' S 150°59 E) have a total area of 114 km2, 38 km2 and 42 km2 respectively (Fig. 1). All three estuaries were declared ‘Recreational Fishing Havens’ in 2002, resulting in the prohibition of commercial fishing. Lake Macquarie and St Georges Basin are classified as ‘wave-dominated’ estuaries which rely predominantly on wind induced-waves for water transport and are characterised by narrow entrances that restrict marine flushing via tidal cycles [25]. Lake Macquarie is the source of cooling water for three power stations located adjacent to the lake and the catchment supports a wide range of land uses from high density urban development, standard residential to agricultural, industrial, mining and conservation areas. In comparison, the St Georges Basin system is relatively undeveloped with 80 % of the area adjacent to the lake consisting of native vegetation [25]. In contrast, Botany Bay is classified as a ‘tide-dominated’ estuary being exposed to ocean swells and having a wide entrance which promotes efficient marine flushing through tidal cycles and wave action. Botany Bay is extensively modified by industrial, urban and port developments and includes shipping 161 terminals, airport runways and large break walls [26].

### Artificial reef construction

Reef location was determined through extensive constraints mapping of existing habitat, suitability of bottom type and depth for safe navigation and consultation with existing user groups. Final reef position within all three estuaries reflects these constraints and the experimental design was required to also accommodate these points.

In each estuary, a total of 180 individual concrete artificial reef units (mini-bay Reef Balls^®^) (Fig. 1E) were divided into six separate replicate artificial reef groups of 30, each defined as an artificial reef set. A total of 540 modules were used to construct 18 artificial reefs across the three estuaries. Construction of the Lake Macquarie artificial reef complex was completed in December 2005, followed by Botany Bay in June 2006 and St Georges Basin in February 2007. Each of the reefs was assigned a designator to identify site, Lake Macquarie (LM) Botany Bay (BB) and St Georges Basin (SGB) and reef type AR1–AR6.

The Lake Macquarie artificial reefs were deployed approximately 180 m apart in depths ranging from 4.5 m to 6 m. The Botany Bay artificial reefs were deployed over a larger area with the distance between reefs ranging from 200–500 m apart in depths ranging from 8 m to 14 m. The close proximity of BBAR1 and BBAR2 to an existing large port revetment wall led to the rapid accumulation of sediment and subsequent burial of these two reefs during the early stages of the project; thought to be a result of increased wave action and sand mobilisation, caused by wave refraction off the wall. As a result, only BBAR3 – BBAR6 have been included in this study. The six reefs deployed at the St Georges Basin site were deployed approximately 400 m apart in depths ranging from 4.5 m to 5.5 m. Three representative natural reef control sites and three sand-flat controls were additionally selected in each estuary.

### Experimental Design and Analysis

Samples were taken on randomly selected days six times per season (3 monthly intervals). To enable a detailed examination of the assemblage structure between artificial reef and natural habitats, while also allowing comparisons between estuaries only a combined summer/autumn season spanning approximately 6 months was analysed in each estuary. (Lake Macquarie: December 2006 to May 2007; Botany Bay & St Georges Basin: December 2007– May 2008). Reef age of the replicate artificial reefs constructed across the three estuaries was consistent in terms of the age and season of the data analysed. However due to variations of deployment times (T0) as a result of disparity in the individual consents for reef construction in each estuary and funding being allocated for reefs construction over a 3 year period, exact deployment and hence reef age could not be controlled.

Replicate samples for each habitat type (artificial reef, natural reef and sand-flat) were collected using three Mono BRUV units. The BRUVs were constructed based on the design of Cappo et al. (2004) and consisted of a stainless steel frame constructed as a mount for the camera and underwater housing. A bait arm (20 mm plastic conduit) extending 1 m from the face of the camera housing supported a plastic container, containing standardised bait (ground chickpea, water and tuna oil), which was replenished prior to every deployment. Initial trials indicated that the standardised mixture provided a constant dispersal over the 30 minute deployment period under a variety of conditions. Cameras were set on ‘short play’ mode and the focus set to ‘manual infinity’. All sampling was done between 08:00 h and 16∶00 h. Length estimates derived from Mono BRUV have been shown to be highly variable and potentially inaccurate. Studies have found that the measurements from Mono BRUV units could be out by as much as 30% and are only useful for looking at broader comparisons such as juvenile verses adult [27]. As a result of this potential bias, no length estimates have been included in this study.

Video analysis was done by the same person [27], [28]. Visibility was estimated directly from the video using the distance to the bait arm (1 m) as a guide. Samples that resulted in incomplete recordings due to technical difficulties or when the estimated visibility was less than 1 m, were rejected from the analysis. Analysis of the video material identified the total number of species observed (species richness – S) as well as the maximum number (maxN) of each species viewed simultaneously during the 30 minute sample period. As this method was used in all locations (artificial reefs and controls) related bias are consistent. Detailed review of the use of maxN as an estimator of relative abundance and its effectiveness have previously been undertaken [29]. Observations from the BRUV video tapes recorded the time to first sighting and max N (the maximum number of individuals of each species observed in one frame over the sampling period) and time of maxN were recorded for all species observed. The use of maxN has been proven to be a reliable and robust method for monitoring fish relative abundance in a variety of inshore marine environments [30]–[32]. The use of maxN over a standardised sample period negates multiple fish counts of individual fish as maxN is a single count taken over the entire 30 min soak period. That is, even if the same fish is recorded by the camera multiple times only a single frame is used as the relative abundance estimate Species diversity was calculated for each sample day within each habitat for all estuaries using the Shannon index (Hs). Mean estimates of sighting frequency were also derived from the BRUVs samples. Sighting frequency is defined as the percentage (%) of the total days sampled in which each species was identified. Sighting frequency was then categorised into four habitat residency groups: permanent species (>75%), frequent species (74.9–30%), scarce species (29.9–10%), and rare (<9.9%) [30].

### Multivariate analysis

A similarity matrix was constructed using fourth-root transformed data and the Bray-Curtis similarity measure for the three combined locations. Non-metric multidimensional scale (nMDS) ordination plot of relative abundance estimates (maxN) was constructed to visually explore patterns in fish assemblages among habitat types between within each estuary [33]. Distance-based permutational multivariate analysis of variance (PERMANOVA) [34] was used to test differences in assemblage structure between estuary and habitat types. The experimental design consisted of three factors: Estuary (random with three levels, Lake Macquarie, Botany Bay, St Georges Basin), Habitat Type (fixed with three levels – artificial reef, natural reef and sand-flat) and Season (fixed with 2 levels summer and autumn). Significant terms and interactions were investigated a posteriori with the PERMANOVA statistic (999 permutations). The combined summer/autumn season (approximately 6 months in each estuary) provided a comparable time period, limiting temporal bias associated with seasonal variation. It should be noted that the time period was selected as it was the oldest comparable data available, representing an artificial reef age of >1 year; which based on fish and benthic assemblage development studies of the artificial reefs [22], [24], represented an assemblage that had progressed through the initial rapid development stage and was approaching early stages of assemblage stability.

Overall patterns of variation in the assemblages identified for each estuary were analysed using metric multidimensional scaling (mMDS). To examine the nature of any significant effects between habitats, canonical analysis of principal coordinates (CAP) [35] was used. Particular species responsible for the observed difference between habitat type and species assemblages were further investigated by calculating correlations with canonical ordination axis. Species showing a Pearsons correlation of r ≥|0.4| were used to identify potential relationships between individual species and the canonical axis are reported. Means (±SEM) for each species (dependent variable) identified by CAP analysis were plotted for each habitat (grouping variable) for each estuary.

### Univariate analysis

Differences between the dependent variables relative abundance 258 (max N); species richness (S) and diversity (Hs) associated with each of the habitat types in each of the estuaries were analysed using a one way analysis of variance. Where significant differences were found, a post-hoc test (Tukey) was performed to further investigate significant differences between categorical predictors.

## Results

### Synopsis

A total of 53 species (representing a combined abundance [MaxN] of 6,853 individual fish) were identified across all three habitats in the three estuaries. The artificial reefs consistently exhibited the greatest number of species when compared to natural rocky reef and sand-flat. Botany Bay had the greatest number of species (43 spp.), regardless of habitat type, with Lake Macquarie and St Georges Basin recording similar species abundance (22 and 16 spp. respectively). Lake Macquarie was the only estuary where all species identified across the habitats were found to reside on the artificial reef. Botany Bay exhibited the greatest number of ‘artificial reef only’ residents, with 13 species identified as exclusively artificial reefs only. Lake Macquarie and St Georges Basin exhibited similar results with 7 and 6 species respectively identified exclusively on the artificial reefs (Table S1 [Appendix]).

### Differences within estuaries

**Figure 2:**
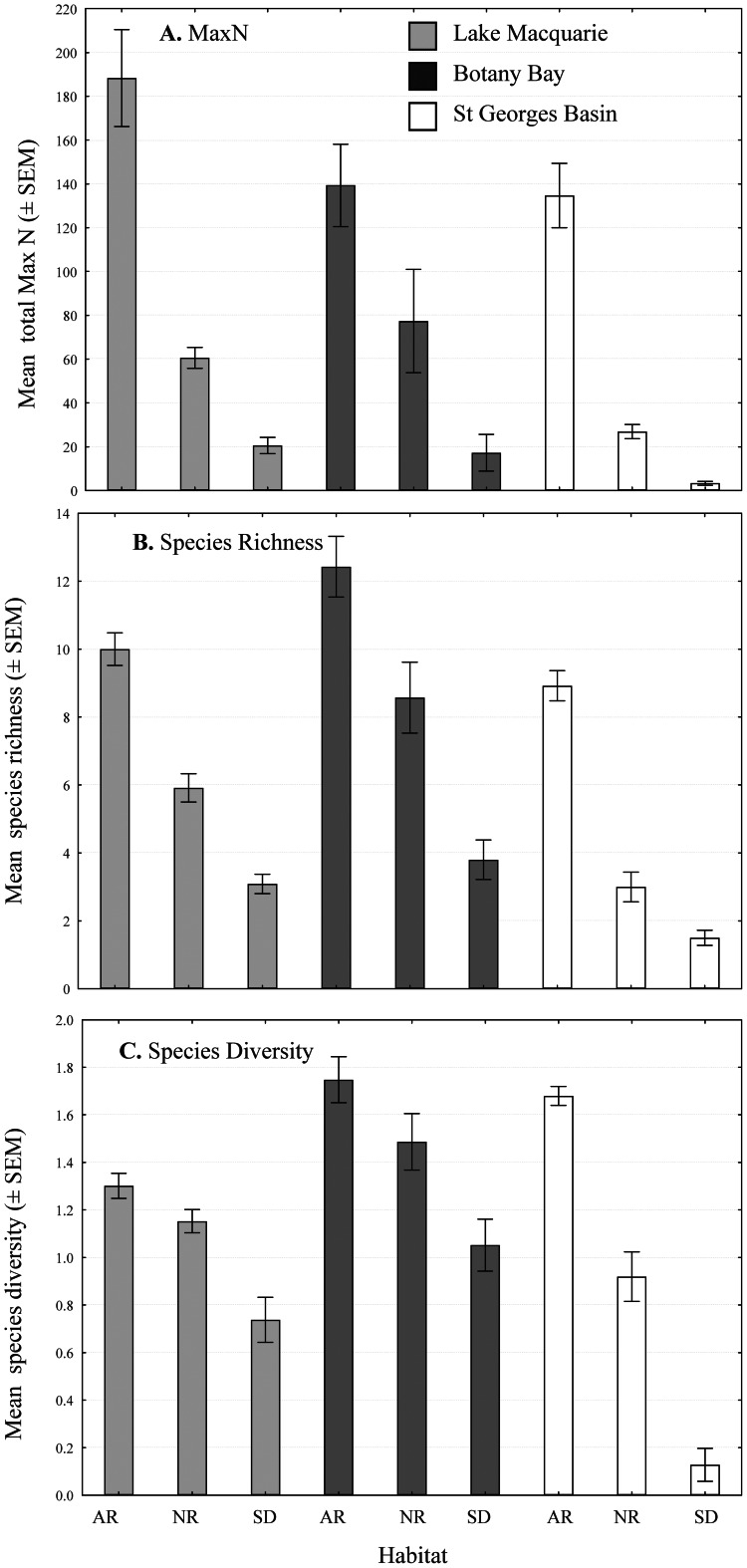
Mean ecological indicators; A. total relative abundance (maxN); B. species richness; and C. species diversity by habitat and location (± SEM) for summer/autumn >12 months of age.

Within estuary comparisons indicated a consistent trend between species response variables and habitat type. Although relative abundance, species richness and diversity were found to be greater at the artificial reef sites than natural reef or sand flat across the three estuaries (P<0.001) (Fig. 2), significance of these comparisons varied (Table 1). Total relative abundance (the combined MaxN for all species identified) on the artificial reefs was consistently observed to be significantly greater 291 (P<0.001) than at the other two habitats, with an average of 188 (±22), 139 (±19) and 135(±15) observed in Lake Macquarie, Botany Bay and St Georges Basin respectively (Fig. 2a). Mean species richness in each location followed a similar pattern, with the artificial reefs significantly more species rich (P<0.05) than at the other two habitats, with an average of 10 (±0.5), 12 (±1) and 9 (±0.5) in Lake Macquarie, Botany Bay and St Georges Basin respectively (Fig. 2b). The artificial reefs in each estuary also demonstrated a more diverse fish assemblage than the other two habitats, with a Shannon diversity index of 1.2 (±0.05), 1.8 (±0.10) and 1.7(±0.04) in Lake Macquarie, Botany Bay and St Georges Basin respectively (Fig. 2c).

**Table 1:** Summary of post-hoc ANOVA (Newman-Keuls) comparisons between A. relative abundance (maxN), B. species richness (S) and C. diversity (Hs) within Lake Macquarie (LM), Botany Bay (BB) and St Georges Basin (SGB).

maxN	LMAR	LMNR	LMSD	BBAR	BBNR	BBSD	SGBAR	SGBNR	SGBSD
LMAR	n/a	**<0.001**	**<0.001**						
LMNR	**<0.001**	n/a	ns						
LMSD	**<0.001**	ns	n/a						
BBAR				n/a	**<0.05**	**<0.001**			
BBNR				**<0.05**	n/a	**<0.05**			
BBSD				**<0.001**	**<0.05**	n/a			
SGBAR							n/a	**<0.001**	**<0.001**
SGBNR							**<0.001**	n/a	ns
SGBSD							**<0.001**	ns	n/a

S									
LMAR	n/a	**<0.001**	**<0.001**						
LMNR	**<0.001**	n/a	**<0.05**						
LMSD	**<0.001**	**<0.05**	n/a						
BBAR				n/a	**<0.001**	**<0.001**			
BBNR				**<0.001**	n/a	**<0.001**			
BBSD				**<0.001**	**<0.001**	n/a			
SGBAR							**n/a**	**<0.001**	**<0.001**
SGBNR							**<0.001**	n/a	ns
SGBSD							**<0.001**	ns	n/a

Hs									
LM AR	n/a	ns	**<0.001**						
LM NR	ns	n/a	**<0.001**						
LM SD	**<0.001**	**<0.001**	n/a						
BB AR				n/a	ns	**<0.001**			
BB NR				ns	n/a	**<0.05**			
BB SD				**<0.001**	**<0.05**	n/a			
SGB AR							n/a	**<0.001**	**<0.001**
SGB NR							**<0.001**	n/a	**<0.001**
SGB SD							**<0.001**	**<0.001**	n/a

Significant results reported in bold.

#### Sighting frequency

**Figure 3:**
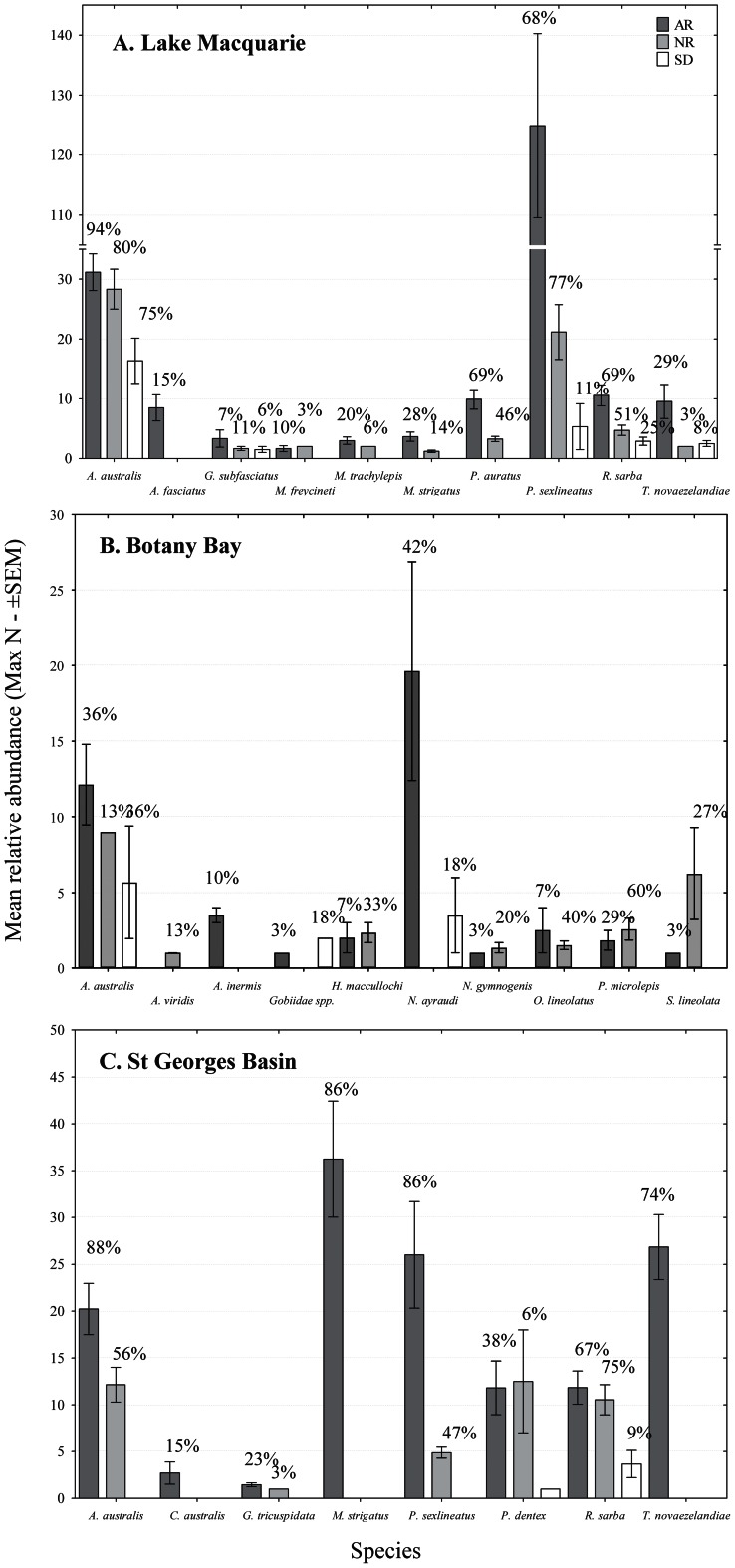
Mean relative abundance (maxN – ±SEM) for species correlated with habitat assemblages by CAP analysis for A. Lake Macquarie, B. Botany Bay, C. St Georges Basin. Frequency of occurrence are given as % above bars.

Only a small number of species were classified as being observed permanently or frequently (sighting frequency >30%) across all estuaries and habitats. More than 80% of species identified were found to be either scarce or rare. On the artificial reefs, the majority of species identified as permanent or frequent residents were found to be sparids or carangids including *Acanthopagrus australis*, *Pagrus auratus, Microcanthus strigatus, Pelates sexlineatus* and *Trachurus novaezelandiae*. A. australis was the only species observed as either a permanent or frequent artificial reef resident in all three estuaries. Other notable sparid species including *P. auratus* and *Rhabdosargus sarba* were also found to be permanent or frequent artificial reef residents in two of the three estuaries sampled (Fig. 3A–C, Table S1 [Appendix]). Species identified with the highest frequency on sand-flat habitat across the three estuaries were also dominated by the sparids *A. australis* and *P. auratus*.

In Lake Macquarie, only *P. sexlineatus* was identified with greater frequency on natural rocky-reef than on either artificial reefs or sand-flat habitats. No species were found to be permanent or frequent reef residents unique to one particular habitat type (Fig. 3A). Botany Bay was the only estuary that exhibited a high sighting frequency for non sparid/carangid species (Fig. 3B). These included *Atypichthys strigatus* on the artificial and natural rocky-reef and *Hypoplectrodes maccullochi, Ophthalmolepis lineolatus* and *Parma microlepis* on the rocky-reef only. Botany Bay was the only estuary where *A. australis* did not dominate sighting frequency on the artificial reef, identified with higher frequency on the sand-flat. In St Georges Basin, *A. australis* and *P. sexlineatus* were found with higher frequency on the artificial reefs when compared to rocky-reef. Conversely, *R. sarba* was identified with greater frequency on the rocky-reef (Fig. 3C).

#### Community variation

**Figure 4:**
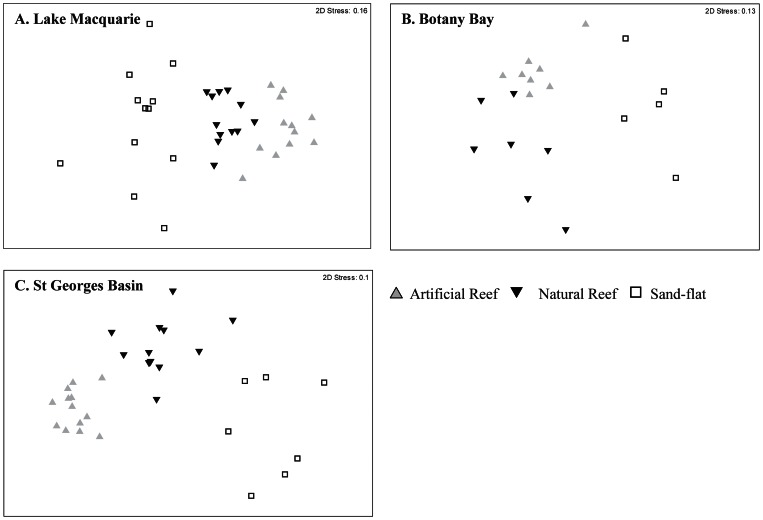
Non-metric multidimensional scale (nMDS) ordination plot of total relative abundance (maxN – forth root transformed) for all species in each habitat type.

The nMDS plot illustrates separation of habitat types in each of the three estuaries (Fig. 4A–C). Results of PERMANOVA analysis showed a significant interaction between Habitat and community structure in the three estuaries (Table 2A–C).

**Table 2:** Summary of results of permutational multivariate analysis of variance (PERMANOVA) dor forth-root transformed data based on Bray-Curtis similarities with two factors: Habitat (artificial reef – AR, natural reef – NR, sand-flat – SD).

A.				
LM – PERMANOVA				
Factor	df	MS	*F*	p
HABITAT	2	8900.4	16.318	**<0.001**
SEASON	1	1509.8	2.7681	**<0.05**
HABITAT × SEASON	2	750.18	1.3754	0.209
Residual	30	545.43		

PAIR-WISE COMPARISONS				
Groups	*t*	*p*		
AR/NR	2.1211	**<0.001**		
AR/SD	2.2972	**<0.001**		
NR/SD	1.9991	**<0.05**		

B.				
BB – PERMANOVA				
Factor	df	MS	*F*	p
HABITAT	2	7129.2	4.5348	**0.001**
SEASON	1	2857.8	1.8178	0.052
HABITAT × SEASON	2	1964.6	1.2496	0.226
Residual	14	1572.1		

C.				
SGB – PERMANOVA				
Factor	df	MS	*F*	p
HABITAT	2	23180	45.332	**<0.001**
SEASON	1	1446.2	2.8282	0.052
HABITAT × SEASON	2	587.14	1.1482	0.355
Residual	32	511.35		

PAIR-WISE COMPARISONS				
Groups	*t*	*p*		
AR/NR	5.6185	**<0.001**		
AR/SD	8.6533	**<0.001**		
NR/SD	5.6554	**<0.001**		

Pairwise comparisons between estuaries. A. lake Macquarie (LM), B. Botany Bay (BB), C. St Georges Basin (SGB). Significant results reported in bold.

**Figure 5:**
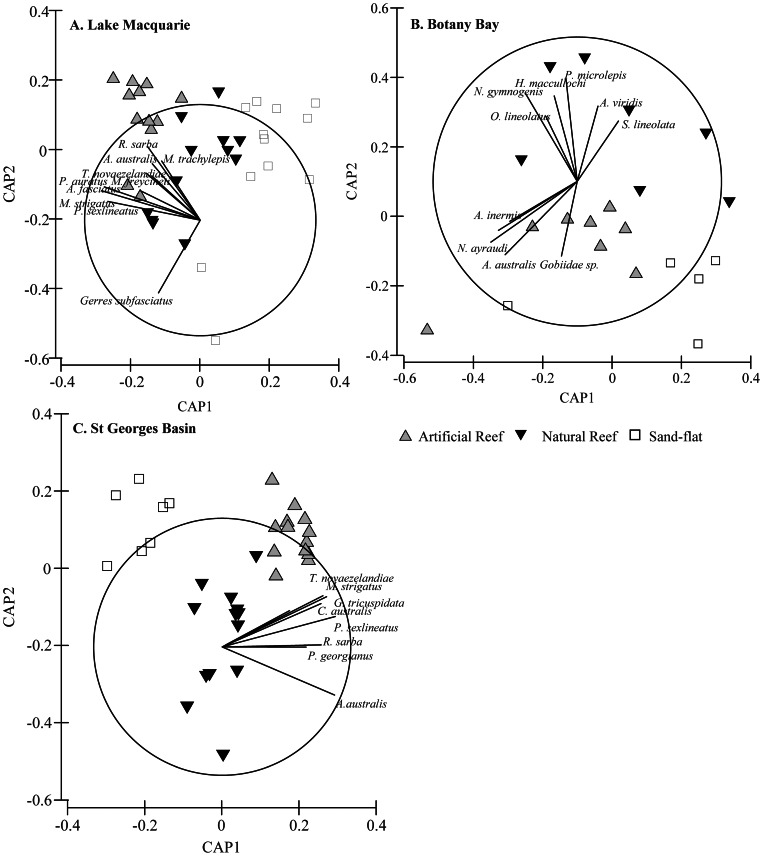
Graphic representation of CAP examining the effects of habitat type and illustrating species responsible for differences in habitat communities.

The a posteriori pair-wise test among levels of the factor Habitat also showed highly significant differences (P<0.001) between all combinations for habitat in all three estuaries. The graphical depiction of CAP correlates (Fig. 5A–C) illustrates the variation in species associations between habitats in each estuary. Canonical analysis of principal coordinates (CAP) revealed significant differences in species between habitat type in all estuaries (Table 3). The vast majority of species across the three estuaries were correlated (r ≤|0.4|) with the artificial reefs. In Lake Macquarie and St Georges Basin three sparids and one carangid were consistently correlated with artificial reefs. *A. australis* was the only species to be correlated with artificial reefs across all three estuaries. Other species of note were *P. sexlineatus* and *M. strigatus*, both found to be positively correlated with artificial reefs in Lake Macquarie and St Georges Basin, but were not found to be significantly correlated with any habitat in Botany Bay. No species were found to be positively correlated to rocky- reef or sand-flat in all three estuaries. Botany Bay was the only estuary where a number of species were found to be correlated with the natural reef. No species were found to be positively correlated with sand-flat habitat in any of the estuaries sampled.

**Table 3:** Results of CAP examining the effects of Habitat on fish assemblages in each estuary – Lake Macquarie (LM), Botany bay (BB), St Georges Basin (SGB).

SITE	m	% Var.	Allocation success (%)				δ^2^	P
			AR	NR	SD	Total		
LM	3	97.22	91.67	100	100	97.22	0.93	**<0.001**
BB	3	90	100	85.71	80	90	0.87	**<0.001**
SGB	4	97.37	100	100	100	100	0.95	**<0.001**

Significant results reported in bold. % Var.  =  percentage of total variation explained by the first m principal coordinates axes; Allocation success  =  percentage of points correctly allocated to each group; δ2 =  squared canonical correlation.

## Discussion

When evaluating artificial reefs, locating comparable natural rocky-reef is practically very difficult [10], [18]. However, if the pre-determined goal of the reefs construction is the addition of reef area that will provide habitat for fish that is comparable to adjacent natural rocky-reef, then reef performance should be evaluated using simultaneous comparisons with the surrounding natural habitats [10]. Further, to understand the interaction between assemblages identified on the artificial reef and surrounding sedimentary habitats, the survey design should include sand-flat; representative of the sedimentary zones surrounding artificial reefs [6]. The artificial reefs studied were constructed in a multi-component design using purpose built concrete modules (Reef Balls^®^) deployed on sand sites that were isolated from natural rocky-reef. This study exclusively used mono BRUV units for data collection. BRUVs as a stand alone method have been shown to be suitable for sampling mobile demersal and semi-pelagic fish associated with artificial reefs with the exception of heavily reef associated cryptic species [36]. Underwater visual census (UVC) has been shown to record significantly greater species richness and diversity. Therefore, BRUV although an effective method for recording species associated with artificial reefs may underestimate cryptic species that are located within the reef structure itself resulting in reduced species richness estimates. Our study found that the artificial reefs consistently exhibited a greater relative abundance, species richness and diversity. As with a number of related studies [12], [19], [37], the artificial reef assemblage was not found to be a direct surrogate for natural rocky-reef as the assemblage varied significantly with those communities identified on the rocky reef and sand-flat.

### Differences within estuaries

Differences in fish communities among estuaries are not surprising. Abiotic factors including reef age, reef size, location (degree of isolation) [10] and differences in topography (the reefs structural complexity) [38]–[40] are all known to contribute to variation in fish assemblagse. However, these differences between habitats and the assemblages they support may also extend within estuarine systems themselves. For example, the physical structure of two distinct artificial habitats (swimming enclosures versus marinas) within the same location have been shown to support different communities within the one estuary [19]. However, these differences were not consistent between locations within the same estuary, indicating that not only structure, but also location, plays a role in structuring the fish community.

Within all three estuaries, higher species richness, diversity and abundances were recorded on the artificial reefs when compared to the adjacent natural habitats, a result that is consistent with a number of related studies [11], [12], [20], [41], [42]. Although the structure of a fish assemblage will differ with reef types, reef sizes and reef locations, it may be fair to assume that separate assemblages located within small-scale geographical locations (i.e. within the same estuary) should be made up of a similar suite of species. Therefore, a comparatively isolated artificial reef fish assemblage, surrounded by large expanses of sand-flat should be made up of a combination of species that are more likely to occur on a range of adjacent natural habitats including rocky-reef, sand-flat and open-water. Inevitably, this will lead to varying, sometimes elevated species richness and diversities when compared to adjacent rocky-reefs. In keeping with this prediction, our study found that although the artificial reef assemblages identified did consist of a similar group of species to those found on the adjacent natural habitats (in some cases located less than 1 km from the reef sites itself), the assemblage did differ significantly. For example, in Lake Macquarie, *T. novaezelandiae* was found in higher abundance and frequency on the sand-flat than on the rocky-reef, but was found to be a key artificial reef associated species. Conversely, *P. auratus* was identified on rocky-reef but not on sand-flat, but was also found to be an important contributor to the artificial reef assemblage. These variations in species proportions between habitats may be in part attributed to the location or isolation of the artificial reefs or indeed the quality of the habitat itself. The patch design and relative isolation of the artificial reefs were vastly different to the fragmented rocky-reef controls; this is likely to have influenced the resultant artificial reef assemblage. Previous studies have found that differences in fish assemblages between artificial and natural reef habitats is in part explained by the movement of post settlement fish on and off an artificial reef [18]. Species such as *A. australis* that dominated the artificial reef community were identified regularly on sand-flat. *A. australis* are common in south-east Australian estuaries [43], known to undertake extensive pre-spawning migrations and are known to inhabit artificial structures in relatively high numbers [19]. The prevalence of *A. australis* on the artificial reefs is a result of the species ability to travel between habitats, often over sand-flat, that may be perceived as a barrier for other less mobile or heavily reef associated species. However it should be noted that this species is also readily found on sand-flat demonstrating its ability to inhabit a range of habitats types. It is known that some reef associated species are capable of moving over bare sand [44], while others are reluctant to cross it [45]–[47], perceived as barriers of variable permeability [46], [48], [49]. Medium-sized (post settlement adult and sub-adult) mobile fish are least influenced by reef isolation or low habitat connectivity [47], [50], [51], with sparids previously shown to cross relatively large expanses of sand where little protection from predation is found [47].

While the location of the artificial reef relative to naturally occurring habitats is a major source of variation, it is unlikely that the location alone of the artificial reefs will dictate the resultant assemblage structure. Species specific behavioural traits such as feeding preferences are also likely to structure the assemblage. Hence, the system is not only being limited by its location, but also by the habitats favourability in terms of feeding preferences. *A. australis* were prevalent on the artificial reefs in high numbers, classified as a permanent artificial reef resident (sighting frequency >75%) in Lake Macquarie and St Georges Basin. This is likely to be a combination of reef location combined with the feeding suitability of the artificial reef itself as the *A. australis* has a preference for feeding in habitats that have a large reef/sand interface [52] as was provided by the patch-like artificial reef groups. Previous studies that have compared a variety of estuarine artificial structures (marinas versus swimming enclosures) in Sydney Harbour found that *A. australis* was more abundant on artificial structures than on natural rocky reef [19]. A possible reason for this observed high abundance was identified as increased access to food, afforded by the ‘edge-to-area’ ratio, typical of small patch artificial reefs. Artificial reefs constructed in isolation may increase the amount of available sand/reef interface, a favoured grazing ground for this species [53]. The results of our study identified two additional sparid species, *P. auratus* and *R. sarba* as additional key contributors to the artificial reef assemblage, whose highly mobile nature and feeding habits similar to that of *A. australis* may have also made the artificial reefs favourable habitat [54], [55]. Mid-water schooling carangids including *T. novaezelandiae* (on the Lake Macquarie and St Georges Basin artificial reefs) and *Pseudocaranx georgianus* (St Georges Basin artificial reef) were correlated with the artificial reefs as a direct result of their transient nature. These species are capable of travelling large distances over reef devoid habitat [56], [57] and it is likely that the location of the artificial reef provided a feeding focal point, as midwater schooling species in general are expected to respond to the overall presence of an artificial reef, rather than being attracted to its fine structure or complexity [39], [40], [58].

The higher abundances and species numbers observed on the artificial reefs as part of this study may have also been as a result of reduced predation pressure related to the reef's location. Reef location has also been linked to top-down ‘predator’ control of community structure [59], with predation pressure found to be higher on larger or more continuous reefs [60], as opposed to smaller isolated ones. For example, a study in the Red Sea that examined whether isolation created differences in fish assemblages on artificial reefs, through changes in predation pressure, reported a positive correlation between species richness and diversity and artificial reef isolation, with resident fish species exhibiting a sharp decline in numbers when the artificial reef (and its inhabitants) were relocated closer to natural reef. Small isolated patch artificial reefs have been shown to attain a higher overall fish diversity than similar sized continuously connected reefs, as lower predation pressure is thought to result in a relatively higher species diversity [61]. Although the sampling method (BRUV) and use of multiple units does address some of the associated bias, the use of BRUV may have the effect of underestimating the abundances and diversity of assemblages associated with continuous natural reef used as a reference point in this study. As fish are likely to be spread over a wider available habitat provide by continuous reef, the likely hood of the fish being attracted to the bait and ultimately within the view of the BRUV is limited in terms of the fishes willingness and ability to move across the fringing habitat and hence being captured by the camera. This bias should be considered in the context of the results provided by our study and the potential underestimation of species numbers and abundances on natural reefs sampled.

The relationship between the range of crevice sizes in a habitat and the sizes of organisms sheltering there has been extensively documented [62] and the functional effect that structurally complex habitats can have on mobile fish is well known [62]. Although the abundance or absence of cover is a key theme related to the effectiveness of artificial reefs, many marine studies have traditionally neglected physical structure and complexity of fish habitat, in turn underestimating the impact of its degradation and simplification for stock sizes [62]. Growth, survival, fecundity, and settlement are usually shaped by food quality and availability and are strongly related to the complexity of the substrate (e.g., as a cover for predators; Caddy 2007). This study would have benefitted considerably with the inclusion of complexity indices as indicators of the habitats ability to maintain viable fish assemblages. Future studies should aim to recognise the importance of such variables, even at fine scales and include then into their subsequent study designs.

### Conclusions

Estuarine artificial reefs show the potential to play a beneficial role in the enhancement of habitats for a range of recreationally important sparid and carangid species. However, these reefs should not be viewed as direct surrogates for natural reef, rather as a hybrid assemblages made up of species that are found on a variety of adjacent natural habitats. Although the assemblage did share many species with adjacent natural habitats, some were identified on the artificial reef alone, resulting in an assemblage that remained distinct by its higher abundances, species richness and diversity. The positive correlation of sparids and carangids to the artificial reef appears to have been as a direct result of their mobility and feeding preferences. Mobility allowed species such as *A. australis* to be able to locate and reside on the artificial reefs in relatively high numbers, while feeding preferences such as increased edge to area ratios may have further attributed to the observed variation in assemblage composition. Our study demonstrated the value in incorporating sand-flat habitat in experimental designs as this helped establish species vagility. The use of stereo-video BRUV units to make accurate determination of fish length (and inferences of biomass) would be beneficial in future. Repeats of this study (or other similar studies using BRUV units) should aim to incorporate stereo-units as the inclusion of biomass estimates; particularly in light of density-dependent habitat selection effects of some species and the resultant effect upon individual fish size and condition [8] would be very useful. It is likely that the future construction of artificial reefs in temperate estuarine systems may provide reef habitat for an abundant, species rich and diverse fish assemblage, however it is unclear as to whether these reef systems will act as fish producers or fish attractors.

## Supporting Information

Table S1 **Elative abundance** (**mean maxN ± SEM**)**, total count and frequency** (**%**) **for all species in A. Lake Macquarie** (**LM**)**, B. Botany Bay** (**BB**) **and C. St Georges Basin** (**SGB**) **by habitat type identified by BRUV002E.**
(XLS)
